# Spatial and temporal distribution of neglected tropical diseases: overlap analysis, Piauí, 2013-2022

**DOI:** 10.1590/S2237-96222026v35e20250373.en

**Published:** 2026-03-16

**Authors:** Lara Beatriz de Sousa Araújo, Roniele Araújo de Sousa, Bruno Guedes Alcoforado Aguiar, Vagner José Mendonça, Olívia Dias de Araújo, Fábio Solon Tajra

**Affiliations:** 1 Universidade Federal do Piauí, Programa de Pós-Graduação em Saúde e Comunidade, Teresina, PI, Brazil; 2 Universidade Federal do Piauí, Centro de Inteligência em Agravos Tropicais Emergentes e Negligenciados, Teresina, PI, Brazil

**Keywords:** Neglected Diseases, Social Determinants of Health, Time Series Studies, Public Health Surveillance, Health Surveillance System, Enfermedades Desatendidas, Determinantes Sociales de la Salud, Estudios de Series Temporales, Vigilancia en Salud Pública, Sistema de Vigilancia Sanitaria

## Abstract

**Objective:**

To analyze the temporal trends and spatial distribution of overlapping neglected tropical diseases in Piauí state between 2013 and 2022.

**Methods:**

This was an ecological, quantitative and analytical time-series study of cases of Chagas disease, leishmaniasis, leprosy and tuberculosis in Piauí during this period. We used data provided by the Piauí State Health Department, taken from the Notifiable Health Conditions Information System, and population data available from the Brazilian Institute of Geography and Statistics. Sociodemographic variables were investigated, and incidence, prevalence, absolute and relative frequencies were calculated, in addition to temporal trend analyses using the Prais-Winsten regression model, with annual percentage change (APV) and 95% confidence intervals (95%CI).

**Results:**

Between 2013 and 2022, 24,234 cases of these diseases were recorded in Piauí, with emphasis on leprosy (46.2%) and tuberculosis (37.4%). Males were the most affected (60.6%), as was the 20-59 years age group (62.5%). Visceral leishmaniasis prevalence was high in children (33.0%). The temporal trend indicated a reduction in the prevalence of visceral leishmaniasis (APC -12.26%; 95%CI -17.49; -6.71) and leprosy (APC -5.67%; 95%CI -9.47; -1.72), as well as an increase in Chagas disease (APC 19.3%; 95%CI 5.09; 35.43) and tuberculosis (APC 2.2%; 95%CI 0.59; 3.84). The spatial analysis indicated that 25% of the municipalities presented overlapping prevalence of at least three diseases, with emphasis on the Entre Rios, Vale do Rio Guaribas and Cocais regions.

**Conclusion:**

Differentiated patterns of occurrence were observed, especially for leprosy and tuberculosis, as were significant variations between health regions, and an increasing trend for two diseases.

## Abstract (es)

Analizar las tendencias temporales y la distribución espacial de las enfermedades tropicales desatendidas con solapamiento en Piauí entre 2013 y 2022.

**Métodos**

Se realizó un estudio ecológico, cuantitativo y analítico de series temporales de casos de enfermedad de Chagas, leishmaniasis, lepra y tuberculosis en Piauí durante este período. Se utilizaron datos proporcionados por la Secretaría de Salud del Estado de Piauí, del Sistema de Información de Enfermedades de Declaración Obligatoria, y datos poblacionales del Instituto Brasileño de Geografía y Estadística. Se investigaron variables sociodemográficas y se calcularon la incidencia, la prevalencia, las frecuencias absolutas y relativas, además de análisis de tendencias temporales mediante el modelo de regresión de Prais-Winsten, con variación porcentual anual (VPA) e intervalos de confianza del 95% (IC95%).

**Resultados**

Entre 2013 y 2022, se registraron 24.234 casos de estas enfermedades en Piauí, con especial atención a la lepra (46,2%) y la tuberculosis (37,4%). Los hombres fueron los más afectados (60,6%) y el grupo de edad de 20 a 59 años (62,5%). La leishmaniasis visceral presentó alta prevalencia en niños (33,0%). La tendencia temporal indicó una reducción en la prevalencia de leishmaniasis visceral (VPA -12,26%; IC95% -27,49; -6,71) y lepra (VPA -5,67%; IC95% -9,47; -1,72), así como un aumento en la enfermedad de Chagas (VPA 19,3%; IC95% 5,09; 35,43) y tuberculosis (VPA 2,2%; IC95% 0,59; 3,84). El análisis espacial indicó que el 25% de los municipios presentó prevalencias superpuestas de al menos tres enfermedades, con énfasis en las regiones de Entre Ríos, Vale do Rio Guaribas y Cocais.

**Conclusión**

Se observaron patrones diferenciados de ocurrencia, especialmente para lepra y tuberculosis, variaciones significativas entre regiones sanitarias y una tendencia creciente para dos enfermedades.

**Ethical aspects**

This research used public domain anonymized databases.

## Introduction 

Neglected tropical diseases represent a heterogeneous group of chronic infections prevalent in tropical and subtropical regions, primarily affecting the most vulnerable populations. Characterized by high morbidity and mortality rates, neglected diseases are marked by a cycle of social and economic exclusion, perpetuated by their close relationship with social and environmental determinants, such as inadequate sanitation, malnutrition and limited access to health services (1).

Although tuberculosis is not traditionally classified as a neglected disease by the World Health Organization, it shares epidemiological and socioeconomic characteristics that justify its inclusion in this group. Tuberculosis disproportionately affects low-income populations and represents a major cause of health inequality, with a high concentration of cases and deaths. This overlapping of factors highlights neglect in combating tuberculosis, aligning it with the profile of neglected diseases (2,3).

Piauí, a state in northeastern Brazil, is notable for its high levels of social vulnerability and high incidence of neglected tropical diseases, such as Chagas disease, leishmaniasis and leprosy. The coexistence of these diseases is influenced by environmental, social and economic factors and is exacerbated by limited access to diagnosis and treatment, placing a strain on the healthcare system (4,5).

The burden of neglected tropical diseases is often underestimated, especially in regions with favorable conditions for their persistence but limited access to healthcare (6). This leads to increased hospitalizations due to diagnosis shortcomings or errors, coinfections, complications and treatment abandonment, overburdening the healthcare system and requiring the intervention of more complex services (7). Despite this, there are still gaps in the literature on the spatiotemporal distribution and hotspots of these diseases, hindering the targeting of effective actions.

In response, the Ministry of Health launched the Healthy Brazil Program in 2023, which includes goals to reduce the incidence of diseases such as tuberculosis and leprosy. In Piauí, the State Plan to Address Neglected Tropical Diseases was launched with a focus on integrating actions to control six diseases (leprosy, tuberculosis, systemic mycoses, Chagas disease, visceral leishmaniasis and tegumentary leishmaniasis). Analyzing their overlap is one of the first steps, as this integrated approach allows for the redirection of appropriate resources and the development of more effective control strategies (4).

As such, this study aims to analyze the temporal trend and spatial distribution of overlapping neglected tropical diseases in Piauí between 2013 and 2022. The analysis was carried out based on a situational assessment of health in the state, adopting a broad and comprehensive approach to the diseases that affect the population and that are covered by the state plan, with the exception of systemic mycoses, due to the lack of official case reporting.

## Methods 

### Design 

This was an ecological, quantitative and analytical time-series study of neglected tropical diseases, focusing on Chagas disease, leishmaniasis (visceral and tegumentary), leprosy and tuberculosis, analyzing temporal trends and describing the spatial distribution and overlap of these diseases.

### Setting, period and participants

The data refer to all cases of the diseases in residents of Piauí from 2013 to 2022. The state of Piauí is located in the Northeast region of Brazil. It had a population of 3,271,199 inhabitants in 2022, a population density of 12.99 inhabitants/km^2^ and a human development index of 0.69 in 2021. In addition, it is composed of 224 municipalities and is divided into 11 health regions, with 98.7% Family Health Strategy coverage (8,9).

### Variables 

The distribution of cases of each disease was analyzed based on sociodemographic variables held on the Notifiable Health Conditions Information System, which included: sex (male and female), age group (0-9 years, 10-19 years, 20-59 years and ≥60 years), municipality of residence, and the 11 health regions of Piauí. Subsequently, in order to assess overlap, the cases of the diseases were recoded and unified for visualization in space-time: if there is at least one case, the code is equal to one; if there are two cases, the code is two, and so on, whereby a municipality can range from zero (no cases of the diseases) to six (presence of all six diseases).

### Data sources 

The data were made available by the Piauí State Health Department (10) in December 2023 via the Notifiable Health Conditions Information System. The following notifiable diseases were included: Chagas disease, visceral leishmaniasis, tegumentary leishmaniasis, leprosy and tuberculosis. Population data for the period analyzed were obtained from the Brazilian Institute of Geography and Statistics.

### Statistical methods

The indicator calculations were performed as follows: a) incidence rate: the number of new cases of the diseases (numerator) was divided by the number of the resident population (denominator), multiplying the result by 100,000; b) prevalence rate: the number of existing cases of the diseases (numerator) was divided by the number of the resident population (denominator), multiplying the result by 100,000.

In order to address duplications or inconsistencies between new and existing cases, despite the database lacking individual identifiers, criteria were adopted based on variables such as sex, age, municipality of residence, date of first symptoms, date of notification and health condition code. In order to identify new case reports, any record the “entry type” of which on the case reporting form contained the code “1 - new case” was considered to be a “new case”. In order to understand the behavior of the diseases over the period analyzed, all confirmed records held on the database were considered to be “existing cases”, regardless of entry type.

Furthermore, relative and absolute frequencies were calculated, in addition to incidence and prevalence rates, using Microsoft Office Excel 2016. We used the data tabulator for Windows (TabWin 415) to calculate the spatial distribution of the occurrence of the diseases.

A temporal trend analysis of the annual prevalence and incidence rates of each disease was performed, using the Prais-Winsten linear regression model. annual percentage change (APC) and its respective 95% confidence intervals (95%CI) were calculated, and the rate trends were interpreted as rising (p-value<0.05 and positive beta), falling (p-value<0.05 and negative beta), or stationary (p-value≥0.05). We used Stata version 14 (StataCorp LP, College Station, USA), taking a 5% level of statistical significance.

## Results 

Between 2013 and 2022, 24,234 cases of neglected tropical diseases were identified in Piauí and analyzed in this study. The most affected groups were males (n=24,234, 60.6%), those aged 20-59 years (n=15,140, ​​62.5%), and those resident in the Entre Rios health region (n=10,846, 44.7%). When stratifying the diseases, we found that leprosy (n=11,210, 46.3%) and tuberculosis (n=9,060, 37.4%) were those that most affected people’s health, accounting for 83.6% of the total.

Furthermore, we found that Chagas disease, unlike the other neglected diseases, most affected females (n=262, 51.1%) and residents of the Vale Rio Guaribas health region (n=165, 32.2%) (Table 1). Regarding visceral leishmaniasis, a high percentage of affected children was found (n=944, 33.0%).

**Table 1:** Neglected tropical disease case distribution, according to sociodemographic characteristics, Piauí, 2013-2022 (n=24,234)

	Diseases					
	Chagas disease	Visceral leishmaniasis	Tegumentary leishmaniasis	Leprosy^a^	Tuberculosis	Total
Variables	n (%)	n (%)	n (%)	n (%)	n (%)	n (%)
Total	513 (100.0)	2,858 (100.0)	644 (100.0)	11,210 (100.0)	9,060 (100.0)	24,234 (100.0)
Sex						
Male	251 (48.9)	1,864 (65.2)	412 (64.0)	6,283 (56.1)	5,866 (64.7)	14,676 (60.6)
Female	262 (51.1)	944 (34.8)	232 (36.0)	4.,26 (43.9)	3,194 (35.3)	9,558 (39.4)
**Age group** (years)						
0-9	37 (7.2)	944 (33)	24 (3.7)	229 (2.0)	161 (1.8)	1,395 (5.8)
10-19	55 (10.7)	320 (11.2)	45 (7.0)	870 (7.8)	447 (4.9)	1,737 (7.2)
20-59	289 (56.3)	1,299 (45.5)	421 (65.4)	7,007 (62.5)	6,124 (67.6)	15,140 (62.5)
≥60	132 (25.7)	295 (10.3)	154 (23.9)	3,104 (27.7)	2,328 (25.7)	6,013 (24.8)
**Health Regions**						
Carnaubais	13 (2.5)	104 (3.6)	18 (2.8)	637 (5.7)	453 (5.0)	1,225 (5.0)
Chapada das Mangabeiras	9 (1.8)	290 (10.1)	45 (7.0)	704 (6.3)	332 (3.7)	1,380 (5.7)
Cocais	123 (24.0)	406 (14.2)	172 (26.7)	852 (7.6)	1,002 (11.1)	2,555 (10.5)
Entre Rios	84 (16.4)	1,166 (40.8)	300 (46.6)	5,061 (45.1)	4,235 (46.7)	10,846 (44.7)
Planície Litorânea	15 (2.9)	219 (7.7)	11 (1.7)	801 (7.1)	1,067 (11.8)	2,113 (8.7)
Serra da Capivara	15 (2.9)	151 (5.3)	10 (1.6)	416 (3.7)	255 (2.8)	847 (3.5)
Tabuleiros do Alto Parnaíba	0 (0.0)	26 (0.9)	22 (3.4)	161 (1.4)	103 (1.1)	312 (1.3)
Vale do Canindé	54 (10.5)	101 (3.5)	4 (0.6)	352 (3.1)	236 (2.6)	747 (3.1)
Vale do Rio Guaribas	165 (32.2)	213 (7.5)	28 (4.3)	1,100 (9.8)	674 (7.4)	2,180 (9.0)
Vale do Sambito	16 (3.1)	46 (1.6)	15 (2.3)	284 (2.5)	252 (2.8)	613 (2.5)
Vale dos Rios Piauí e Itaueiras	19 (3.7)	136 (4.8)	19 (3.0)	842 (7.5)	451 (5.0)	1,467 (6.0)

^a^One (1) value with no information on the “sex” variable.

**Figure 1:**
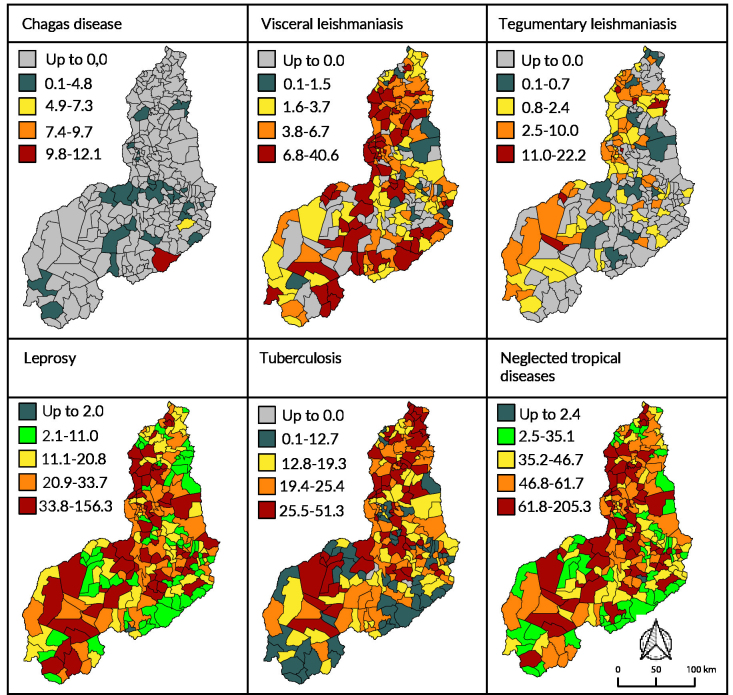
Distribution of average prevalence of neglected tropical diseases. Piauí, 2013-2022

**Figure 2:**
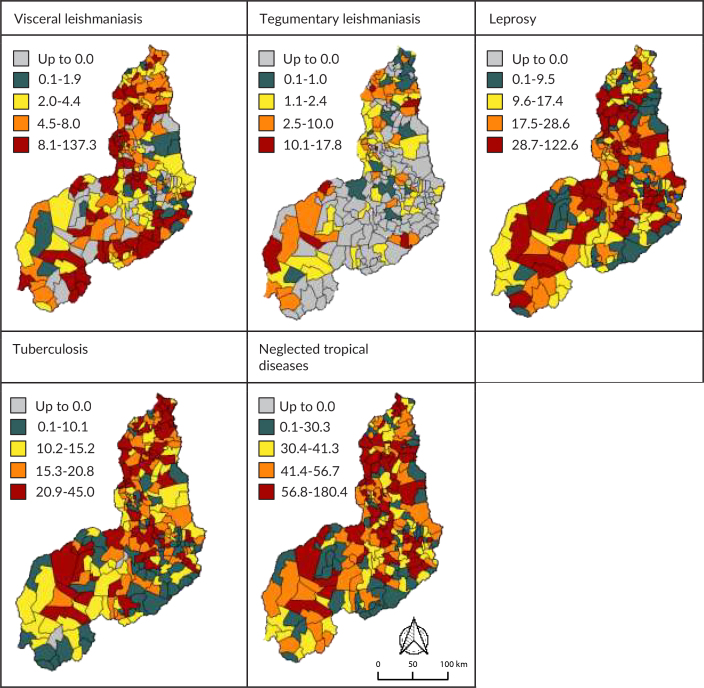
Distribution of average incidence of neglected tropical diseases. Piauí, 2013-2022

Figures 1 and 2 show the spatial distribution of prevalence and incidence rates, respectively, for the disease in Piauí. When examining the map legends for each disease, variations in prevalence and incidence were identified across municipalities. For both indicators, leprosy and tuberculosis presented higher values ​​with distinct distributions across all health regions and across the 224 municipalities.

Average prevalence (Figure 1) of Chagas disease was highest in Dom Inocêncio, with a rate of 12.1 cases/100,000 inhabitants. Visceral leishmaniasis had high average prevalence (from 6.8 to 40.8 cases/100,000 inhabitants) in 56 (25.0%) municipalities, mostly distributed in the Entre Rios region (32.1%). Tegumentary leishmaniasis had high mean prevalence (from 10.1 to 22.2 cases/100,000 inhabitants) in six (2.7%) municipalities, with the highest distribution being found in the Cocais region (33.3%).

It was also possible to visualize (Figure 1) average prevalence classified as high: for leprosy (from 33.8 to 156.3 cases/100,000 inhabitants), identified in 55 (24.6%) municipalities, with the majority in Entre Rios (21.8%); and for tuberculosis (from 25.5 to 51.3 cases/100,000 inhabitants), in 56 (25.0%) municipalities and most affected in the Entre Rios region (14.3%). In addition, 56 (25.0%) municipalities had high average prevalence (from 61.8 to 205.3 cases/100,000 inhabitants) for all the neglected diseases in this study, with a significant concentration in the Entre Rios region (21.4%).

Figure 2 shows the average incidence of the tropical diseases in Piauí. We identified that 54 (24.1%) of the municipalities had high average incidence (from 8.1 to 137.3 cases/100,000 inhabitants) for visceral leishmaniasis, with the highest concentration in the Entre Rios region (24.1%). Tegumentary leishmaniasis had high average incidence (from 6.8 to 40.6 cases/100,000 inhabitants) in 16 (7.1%) municipalities, concentrated in the Cocais (31.3%) and Chapada das Mangabeiras (25.0%) regions.

In the case of leprosy, average incidence classified as high (from 18.7 to 122.6 cases/100,000 inhabitants) occurred in 106 (47.3%) municipalities, predominantly located in the Entre Rios region (19.8%). Tuberculosis, in turn, had high average incidence (from 20.9 to 45.0 cases/100,000 inhabitants) in 56 (25.0%) municipalities, concentrated in the same region (17.9%). Regarding the neglected diseases overall, 56 (25.0%) municipalities had high average incidence (from 56.8 to 180.4 cases/100,000 inhabitants), also with greater emphasis in the Entre Rios region (19.6%) (Figure 2).

The aggregate group of neglected tropical diseases showed a temporal reduction in prevalence rates (APC -2.79%; 95%CI -5.24; -0.28), whereby visceral leishmaniasis (APC -12.26%; 95%CI -27.49; -6.71) and leprosy (APC -5.67%; 95%CI -9.47; -1.72) were the only diseases that also showed a decrease in rates. In contrast, the prevelance trends of Chagas disease (APC 19.30%; 95%CI 5.09; 35.43) and tuberculosis (APC 2.20%; 95%CI 0.59; 3.84) increased (Table 2).

**Table 2:** Annual percentage change (APC) and 95% confience interval (95%CI) of neglected tropical disease prevalence and incidence (100,000 people). Piauí, 2013-2022

	APC (95%CI)	p-value	Trend
Prevalence			
Neglected tropical diseases	-2.79 (-5.24; - 0.28)	0.034	Falling
Chagas disease	19.3 (5.09; 35.43)	0.012	Rising
Visceral leishmaniasis	-12.26 (-17.49; -6.71)	<0.001	Falling
Tegumentary leishmaniasis	-1.08 (-17.58; 18.72)	0.894	Stationary
Leprosy	-5.67 (-9.47; -1.72)	0.011	Falling
Tuberculosis	2.2 (0.59; 3.84)	0.014	Rising
Incidence			
Neglected tropical diseases	-2.96 (-5.25; 0.62)	0.02	Falling
Chagas disease	-	-	–
Visceral leishmaniasis	-7.88 (-11.18; -4.46)	<0.001	Falling
Tegumentary leishmaniasis	-1.26 (-15.46; 15.31)	0.855	Stationary
Leprosy	-5.75 (-9.65; -1.69)	0.012	Falling
Tuberculosis	1.57 (-0.16; 3.33)	0.069	Stationary

When examining the incidence rates (Table 2), we also identified that there was a reduction in the aggregate group of neglected tropical diseases (APC -2.96%; 95%CI -5.25; -0.62), as well as in visceral leishmaniasis (APC -7.88%; 95%CI -11.18; -4.46) and leprosy (APC -5.75%; 95%CI -9.65; -1.69).

**Figure 3:**
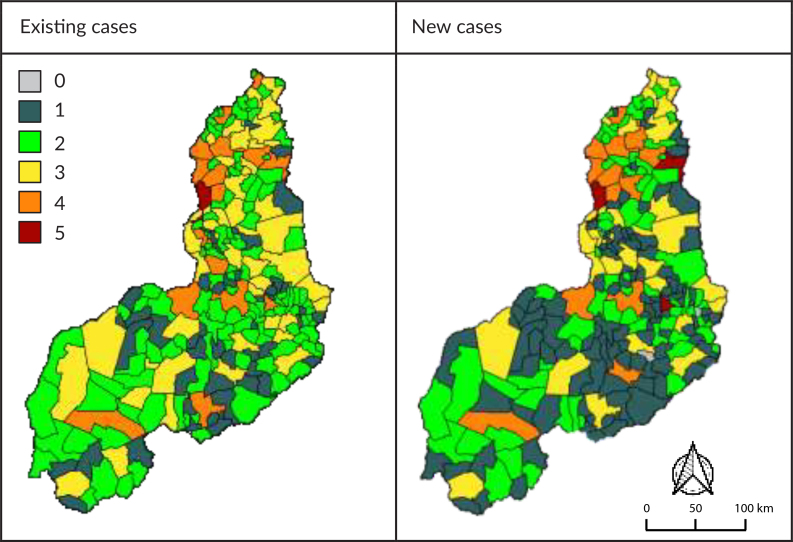
Overlapping of occurrence of types of neglected tropical diseases. Piauí, 2013-2022

Figure 3 presents the average of the time series for the number of diseases that overlap in the municipalities of Piauí. This simple overlap can be visualized both for existing cases of the diseases (prevalence) (3A) and for new cases (incidence) (3B).

Regarding existing cases (3A), only Teresina presented overlapping cases of the five diseases addressed in this study. In contrast, Bela Vista do Piauí and Miguel Leão were classified as silent municipalities, that is, those that received a zero score in the overlapping disease groups. In Figure 3B, which considers new cases, it was possible to verify that Teresina, Picos and Pedro II were the municipalities that presented overlapping cases of all the diseases addressed in this study. On the other hand, Bela Vista do Piauí, Miguel Leão and Vila Nova do Piauí were classified as silent municipalities.

## Discussion 

The analysis of the data from 2013 to 2022 identified specific patterns in the occurrence of neglected tropical diseases in Piauí, characterized by a high concentration of cases and heterogeneous distribution across health regions. The findings indicate that leprosy and tuberculosis stand out as the most prevalent diseases, while Chagas disease and tuberculosis showed rising prevalence rates throughout the period analyzed. The overlap of multiple neglected diseases in certain municipalities, especially the state capital, Teresina, and in regions such as Entre Rios and Vale do Rio Guaribas, highlights areas of greater epidemiological complexity within Piauí.

The epidemiological profile found, with greater incidence among males and those aged 20–59, highlights national patterns of neglected diseases, which often point to greater occupational exposure and risk behaviors among men of working age. The Neglected Tropical Diseases Epidemiological Bulletin, published by the Ministry of Health in 2024, as well as some national and international studies, for example, corroborate this trend of higher incidence among men, which may reflect differences in seeking healthcare. The concentration of cases in the Entre Rios health region, which includes Teresina, can be attributed to its higher population density and the state capital’s role as a referral center for diagnosis and treatment, attracting patients from various locations (5,6,11-13).

The predominance of leprosy and tuberculosis in this study reiterates the persistence of these challenges in Piauí. Corroborating the findings of this investigation, recent studies on Piauí reveal the persistence of high indicators for these diseases, with detection rates consistent with the endemicity scenario identified in this study. Similarly, studies on tuberculosis in Piauí also point to the need for continued attention. Although the state has demonstrated progress with indicators in recent years, identification of areas where leprosy and tuberculosis are endemic highlights the need for more effective strategies, especially given the strong association of these diseases with poverty, social inequalities and barriers to accessing health services. The spatial distribution of these cases, in both urban and rural areas, reinforces the hypothesis that persistence of these diseases is linked not only to biological factors but also to social and structural determinants (14-19).

The epidemiological profile of Chagas disease, which has been shown to be more prevalent in the Guaribas River Valley region, is relevant. The rising prevalence trend of this disease may be related to improved epidemiological surveillance and diagnostic capacity, especially for the chronic phase of the disease. This fact highlights the importance of careful temporal analyses when interpreting mandatory reporting data. The higher incidence found among women, a finding that differs from the profile of other health conditions, is a pattern that can also be observed in other investigations. These patterns suggest that factors such as household exposure, increased screening in specific contexts, such as prenatal care or other specific phases of care, and seeking health services may be involved (20-23).

The high visceral leishmaniasis prevalence found among children is another relevant finding, consistent with previous studies conducted in Piauí. Childhood vulnerability to the disease is often attributed to greater exposure to the vector in domestic environments and an immature immune system. The presence of silent municipalities (with no recorded cases) for some diseases, such as leishmaniasis, raises concerns about underreporting or underdiagnosis, which can distort the true magnitude of the disease and impact the effectiveness of surveillance and control efforts. This may be related to insufficient resources, infrastructure, or technical training for identification and adequate reporting in some locations (14,24,25).

The identification of municipalities with overlapping multiple diseases, especially Teresina, Picos and Pedro II, highlights the complexity of the epidemiological scenario in Piauí. The concentration of neglected diseases in the Entre Rios health region, which includes the state capital and is a referral hub for neighboring states, suggests health service overloading. Although Teresina has more infrastructure, high patient demand may compromise the response capacity and effectiveness of surveillance and treatment efforts in the region. Spatial and temporal analysis, by identifying critical areas and disease trends, is essential for planning and resource allocation, contributing to more effective performance of the Brazilian Unified Health System (26,27).

The downward trend in the prevalence and incidence rates of the neglected diseases group, as well as visceral leishmaniasis and leprosy, indicates that control and early diagnosis measures are producing positive results, albeit unevenly. In contrast, the increase found in the Chagas disease and tuberculosis rates requires reassessment of current strategies and adaptation to changing epidemiological profiles.

This study highlights the importance of intersectoral collaboration and implementation of public policies that go beyond the health sector in order to address neglected tropical diseases. The Healthy Brazil Program, launched by the Ministry of Health in 2023, exemplifies a promising approach by integrating interministerial actions aimed at improving the living and working conditions of the community. Implementation of regional and interministerial strategies, as proposed by this initiative, is crucial to mitigating the impact of these diseases and promoting health equity in Piauí. Strengthening the Primary Care network and decentralizing care are essential to ensuring universal access to diagnosis and treatment, especially in the most vulnerable areas.

This study has some limitations, since use of secondary data can lead to underreporting and duplicate records, which can introduce biases into calculations and compromise the accuracy of incidence and prevalence estimates. Spatial analysis, in turn, may not fully capture local transmission dynamics, especially in municipalities with lower epidemiological surveillance capacity. Despite these limitations, the data analyzed in this research provide a relevant overview of the burden and distribution of neglected tropical diseases in the state of Piauí, thus supporting strategic public health actions.

## Data Availability

The complete data set used in this research is available in the Open Science Framework (OSF) repository, available at: https://osf.io/38u5y/ (28).
