# Supplementary material for: Spatial and temporal distribution of neglected tropical diseases: overlap analysis, Piauí, 2013-2022
Source: Epidemiol Serv Saude. 2026 Mar 16;35:e20250373. doi: 10.1590/S2237-96222026v35e20250373.en (PMC12995279; doi:10.1590/S2237-96222026v35e20250373.en)
Supplement: Translation

# Translation: Distribuição espacial e temporal das doenças tropicais negligenciadas: análise de sobreposição, Piauí, 2013-2022

**DOI:** 10.1590/S2237-96222026v35e20250373.pt

**Authors:** Lara Beatriz de Sousa Araújo, Roniele Araújo de Sousa, Bruno Guedes Alcoforado Aguiar, Vagner José Mendonça, Olívia Dias de Araújo, Fábio Solon Tajra

**Affiliations:** 1 Universidade Federal do Piauí, Programa de Pós-Graduação em Saúde e Comunidade, Teresina, PI, Brasil; 2 Universidade Federal do Piauí, Centro de Inteligência em Agravos Tropicais Emergentes e Negligenciados, Teresina, PI, Brasil

**Keywords:** Doenças Negligenciadas, Determinantes Sociais da Saúde, Estudos de Séries Temporais, Vigilância em Saúde Pública, Sistema de Vigilância em Saúde

## Abstract

**Objetivo:**

Analisar a tendência temporal e a distribuição espacial das doenças tropicais negligenciadas com sobreposição no Piauí entre 2013 e 2022.

**Métodos:**

Estudo ecológico de série temporal, quantitativo e analítico dos casos de doença de Chagas, leishmanioses, hanseníase e tuberculose, nesse período, no Piauí. Utilizaram-se dados disponibilizados pela Secretaria do Estado de Saúde do Piauí, a partir do Sistema de Informações de Agravos de Notificação e dados populacionais do Instituto Brasileiro de Geografia e Estatística. Investigaram-se variáveis sociodemográficas e calcularam-se incidência, prevalência, frequências absolutas e relativas, além de análises de tendência temporal utilizando o modelo de regressão de Prais-Winsten, com variação percentual anual (VPA) e intervalos de confiança de 95% (IC95%).

**Resultados:**

Entre 2013 e 2022, foram registrados 24.234 casos dessas doenças no Piauí, com destaque para hanseníase (46,2%) e tuberculose (37,4%). O sexo masculino foi o mais afetado (60,6%) e a faixa etária de 20-59 anos (62,5%). A leishmaniose visceral apresentou alto percentual em crianças (33,0%). A tendência temporal indicou redução da prevalência para leishmaniose visceral (VPA -12,26%; IC95% -17,49; -6,71) e hanseníase (VPA -5,67%; IC95% -9,47; -1,72), além de aumento para doença de Chagas (VPA 19,3%; IC95% 5,09; 35,43) e tuberculose (VPA 2,2%; IC95% 0,59; 3,84). A análise espacial apontou que 25% dos municípios apresentaram sobreposição de pelo menos três agravos, destacando-se as regiões Entre Rios, Vale do Rio Guaribas e Cocais.

**Conclusão:**

Observaram-se padrões diferenciados de ocorrência, especialmente para a hanseníase e a tuberculose, variações relevantes entre as regiões de saúde e tendência crescente para dois agravos.

**Aspectos éticos**

Esta pesquisa utilizou bancos de dados de domínio público e anonimizados.

## Introdução 

As doenças tropicais negligenciadas representam um grupo heterogêneo de infecções crônicas, prevalentes em regiões tropicais e subtropicais, que afetam primariamente as populações mais vulnerabilizadas. Caracterizadas por elevadas taxas de morbimortalidade, as doenças negligenciadas são marcadas por um ciclo de exclusão social e econômica, perpetuado pela sua estreita relação com determinantes sociais e ambientais, como saneamento inadequado, má nutrição e limitado acesso a serviços de saúde (1). 

Embora a tuberculose não seja tradicionalmente classificada como uma doença negligenciada pela Organização Mundial da Saúde, ela compartilha características epidemiológicas e socioeconômicas que justificam sua consideração nesse grupo. A doença afeta desproporcionalmente populações de baixa renda e representa uma das principais causas de desigualdade em saúde, com alta concentração de casos e óbitos. Essa sobreposição de fatores evidencia a negligência no enfrentamento da tuberculose, alinhando-a ao perfil das doenças negligenciadas (2,3).

O Piauí, estado do Nordeste brasileiro, destaca-se por seus altos índices de vulnerabilidade social e pela alta incidência de doenças tropicais negligenciadas, como doença de Chagas, leishmanioses e hanseníase. A coexistência desses agravos é influenciada por fatores ambientais, sociais e econômicos e é acentuada pela limitação no acesso ao diagnóstico e ao tratamento, sobrecarregando o sistema de saúde (4,5). 

A carga das doenças tropicais negligenciadas é frequentemente subestimada, especialmente em regiões com condições favoráveis à sua manutenção e dificuldades no acesso à saúde (6). Isso leva ao aumento das hospitalizações devido a falhas ou a erros de diagnóstico, coinfecções, complicações e abandono do tratamento, sobrecarregando o sistema de saúde e exigindo a intervenção de serviços mais complexos (7). Mesmo assim, ainda existem lacunas na literatura sobre a distribuição espaço-temporal e as áreas críticas dessas doenças, dificultando o direcionamento de ações eficazes.

Em resposta, o Ministério da Saúde lançou, em 2023, o Programa Brasil Saudável, que inclui metas para reduzir a incidência de doenças como a tuberculose e a hanseníase. No Piauí, o Plano Estadual de Enfrentamento das Doenças Tropicais Negligenciadas foi iniciado com foco na integração de ações para o controle de seis agravos (hanseníase, tuberculose, micoses sistêmicas, doença de Chagas, leishmaniose visceral e leishmaniose tegumentar), sendo a análise da sobreposição deles uma das primeiras etapas, pois essa abordagem integrada permite um redirecionamento de recursos apropriados e o desenvolvimento de estratégias mais efetivas de controle (4).

Nesse sentido, este estudo tem como objetivo analisar a tendência temporal e a distribuição espacial das doenças tropicais negligenciadas com sobreposição no Piauí entre 2013 e 2022. A análise foi realizada a partir de uma avaliação situacional da saúde no estado, adotando uma abordagem ampliada e abrangente sobre os agravos que afetam a população e que estão contemplados no plano estadual, com exceção das micoses sistêmicas, devido à ausência de notificação oficial.

## Métodos 

### Delineamento do estudo

Trata-se de um estudo ecológico de série temporal, quantitativo e analítico acerca de casos de doenças tropicais negligenciadas, com foco sobre a doença de Chagas, as leishmanioses (visceral e tegumentar), a hanseníase e a tuberculose, analisando a tendência temporal e descrevendo a distribuição espacial e a sobreposição das doenças.

### Contexto, período e participantes

Os dados referem-se a todos os casos das doenças em residentes do Piauí de 2013 a 2022. O Piauí localiza-se na região Nordeste do Brasil, contava com uma população de 3.271.199 pessoas em 2022, uma densidade demográfica de 12,99 habitantes/km^2^ e um índice de desenvolvimento humano de 0,69 em 2021. Além disso, é composto por 224 municípios e está dividido em 11 regiões de saúde, com uma cobertura pela Estratégia Saúde da Família de 98,7% (8,9).

### Variáveis 

Foi analisada a distribuição dos casos de cada agravo a partir das variáveis sociodemográficas contidas no Sistema de Informações de Agravos de Notificação, que incluíam: sexo (masculino e feminino), faixa etária em anos (0-9 anos, 10-19 anos, 20-59 anos e ≥60 anos), município de residência e as 11 regiões de saúde do Piauí. Posteriormente, para avaliar a sobreposição, os casos das doenças foram recodificados e unificados para visualização no espaço-tempo: se há pelo menos um caso, o código é igual a um; se há dois casos, o código é dois, e assim sucessivamente, com um município podendo variar de zero (nenhum caso dos agravos) até seis (presença dos seis agravos).

### Fonte dos dados

Os dados foram disponibilizados pela Secretaria de Estado da Saúde do Piauí (10), em dezembro de 2023, por meio do Sistema de Informações de Agravos de Notificação. Foram incluídas as seguintes doenças de notificação compulsória: doença de Chagas, leishmaniose visceral, leishmaniose tegumentar, hanseníase e tuberculose. Os dados populacionais referentes ao período analisado foram extraídos do Instituto Brasileiro de Geografia e Estatística.

### Métodos estatísticos

Os cálculos dos indicadores foram feitos da seguinte forma: a) taxa de incidência: foi dividido o número de casos novos dos agravos (numerador) pelo número da população residente (denominador), multiplicando o resultado por 100 mil; b) taxa de prevalência: foi dividido o número de casos existentes dos agravos (numerador) pelo número da população residente (denominador), multiplicando o resultado por 100 mil.

Para o tratamento de duplicações ou inconsistências de casos novos e existentes, apesar de a base de dados não apresentar identificadores individuais, foram adotados critérios com base em variáveis de sexo, idade, município de residência, data dos primeiros sintomas, data de notificação e código do agravo. A fim de identificar notificações de novos casos, foi considerado “caso novo” todo registro cujo campo “modo de entrada” da ficha de notificação apresentava o código “1 – caso novo”. Para compreender o comportamento dos agravos ao longo do período analisado, foram considerados “casos existentes” todos os registros confirmados presentes na base de dados, independentemente do modo de entrada.

Ademais, foram calculadas as frequências relativas e absolutas, além das taxas de incidência e de prevalência, utilizando o software Microsoft Office Excel 2016. Para a distribuição espacial da ocorrência dos agravos, foi utilizado o tabulador de dados para Windows (TabWin 415).

Aplicou-se a análise de tendência temporal das taxas anuais de prevalência e de incidência de cada agravo e utilizou-se o modelo de regressão linear de Prais-Winsten. A variação percentual anual (VPA) e os seus respectivos intervalos de confiança de 95% (IC95%).foram calculados, e a tendência das taxas foi interpretada como crescente (p-valor<0,05 e beta positivo), decrescente (p-valor<0,05 e beta negativo) e estável (p-valor≥0,05). Utilizou-se o programa Stata versão 14 (StataCorp LP, College Station, EUA) e considerou-se o nível de significância estatística a 5%.

## Resultados 

De 2013 a 2022, foram identificados 24.234 casos de doenças tropicais negligenciadas no Piauí, as quais foram analisadas neste estudo. Os grupos mais acometidos foram as pessoas do sexo masculino (n=24.234, 60,6%), com faixa etária de 20-59 anos (n=15.140, 62,5%) e residentes da região de saúde Entre Rios (n=10.846, 44,7%). Ao estratificar as doenças, verificou-se que a hanseníase (n=11.210, 46,3%) e a tuberculose (n=9.060, 37,4%) foram as que mais afetaram a saúde das pessoas, representando 83,6% do total. 

Além disso, foi verificado que a doença de Chagas, diferentemente das outras doenças negligenciadas, acometeu mais as pessoas do sexo feminino (n=262, 51,1%) e residentes da região de saúde do Vale Rio Guaribas (n=165, 32,2%) (Tabela 1). Já quanto à leishmaniose visceral, foi percebido um alto percentual de crianças acometidas (n=944, 33,0%).

**Tabela 1:** Distribuição dos casos de doenças tropicais negligenciadas, segundo características sociodemográficas. Piauí, 2013-2022 (n=24.234)

	Agravos					
	Doença de Chagas	Leishmaniose visceral	Leishmaniose tegumentar	Hanseníase^a^	Tuberculose	Total
Variáveis	n (%)	n (%)	n (%)	n (%)	n (%)	n (%)
Total	513 (100,0)	2.858 (100,0)	644 (100,0)	11.210 (100,0)	9.060 (100,0)	24.234 (100,0)
Sexo						
Masculino	251 (48,9)	1.864 (65,2)	412 (64,0)	6.283 (56,1)	5.866 (64,7)	14.676 (60,6)
Feminino	262 (51,1)	944 (34,8)	232 (36,0)	4.926 (43,9)	3.194 (35,3)	9.558 (39,4)
**Faixa etária** (anos)						
0-9	37 (7,2)	944 (33)	24 (3,7)	229 (2,0)	161 (1,8)	1.395 (5,8)
10-19	55 (10,7)	320 (11,2)	45 (7,0)	870 (7,8)	447 (4,9)	1.737 (7,2)
20-59	289 (56,3)	1.299 (45,5)	421 (65,4)	7.007 (62,5)	6.124 (67,6)	15.140 (62,5)
≥60	132 (25,7)	295 (10,3)	154 (23,9)	3.104 (27,7)	2.328 (25,7)	6.013 (24,8)
**Regiões de saúde**						
Carnaubais	13 (2,5)	104 (3,6)	18 (2,8)	637 (5,7)	453 (5,0)	1.225 (5,0)
Chapada das Mangabeiras	9 (1,8)	290 (10,1)	45 (7,0)	704 (6,3)	332 (3,7)	1.380 (5,7)
Cocais	123 (24,0)	406 (14,2)	172 (26,7)	852 (7,6)	1.002 (11,1)	2.555 (10,5)
Entre Rios	84 (16,4)	1.166 (40,8)	300 (46,6)	5.061 (45,1)	4.235 (46,7)	10.846 (44,7)
Planície Litorânea	15 (2,9)	219 (7,7)	11 (1,7)	801 (7,1)	1.067 (11,8)	2.113 (8,7)
Serra da Capivara	15 (2,9)	151 (5,3)	10 (1,6)	416 (3,7)	255 (2,8)	847 (3,5)
Tabuleiros do Alto Parnaíba	0 (0,0)	26 (0,9)	22 (3,4)	161 (1,4)	103 (1,1)	312 (1,3)
Vale do Canindé	54 (10,5)	101 (3,5)	4 (0,6)	352 (3,1)	236 (2,6)	747 (3,1)
Vale do Rio Guaribas	165 (32,2)	213 (7,5)	28 (4,3)	1.100 (9,8)	674 (7,4)	2.180 (9,0)
Vale do Sambito	16 (3,1)	46 (1,6)	15 (2,3)	284 (2,5)	252 (2,8)	613 (2,5)
Vale dos Rios Piauí e Itaueiras	19 (3,7)	136 (4,8)	19 (3,0)	842 (7,5)	451 (5,0)	1.467 (6,0)

^a^Um (1) valor sem informação na variável “sexo”.

As Figuras 1 e 2 apresentam a distribuição espacial das taxas de prevalência e de incidência, respectivamente, dos agravos no território piauiense. Observando-se a legenda nos mapas de cada agravo, identificaram-se variações de prevalência e de incidência nos municípios e que, em ambos os indicadores, a hanseníase e a tuberculose apresentaram maiores valores com distribuição distinta em todas as regiões de saúde e nos 224 municípios.

A média de prevalência (Figura 1) da doença de Chagas foi maior em Dom Inocêncio, com a taxa de 12,1 casos/100 mil habitantes. A leishmaniose visceral apresentou média de prevalência elevada (6,8 a 40,8 casos/100 mil habitantes) em 56 (25,0%) municípios, distribuídos em sua maioria na região Entre Rios (32,1%). Já a leishmaniose tegumentar apresentou elevada média de prevalência (10,1 a 22,2 casos/100 mil habitantes) em seis (2,7%) dos municípios, com maior distribuição na região Cocais (33,3%).

**Figura 1:** Distribuição da média da prevalência das doenças tropicais negligenciadas. Piauí, 2013-2022

Também foi possível visualizar (Figura 1) média de prevalência classificada como alta: na hanseníase (33,8 a 156,3 casos/100 mil habitantes), identificada em 55 (24,6%) municípios, com maioria em Entre Rios (21,8%); e na tuberculose (25,5 a 51,3 casos/100 mil habitantes), em 56 (25,0%) municípios e mais acometidos na região Entre Rios (14,3%). Além disso, para todas as doenças negligenciadas deste estudo, 56 (25,0%) municípios tiveram média de prevalência elevada (61,8 a 205,3 casos/100 mil habitantes), com concentração importante na região Entre Rios (21,4%).

A Figura 2 apresenta a média da incidência das doenças tropicais no Piauí. Foi identificado que 54 (24,1%) dos municípios tiveram média de incidência elevada (8,1 a 137,3 casos/100 mil habitantes) para leishmaniose visceral, com maior concentração na região Entre Rios (24,1%). A leishmaniose tegumentar apresentou uma alta média de incidência (6,8 a 40,6 casos/100 mil hab.) em 16 (7,1%) municípios, concentrados nas regiões Cocais (31,3%) e Chapada das Mangabeiras (25,0%). 

**Figura 2:** Distribuição da média da incidência das doenças tropicais negligenciadas. Piauí, 2013-2022

No caso da hanseníase, a média de incidência classificada como alta (18,7 a 122,6 casos/100 mil habitantes) ocorreu em 106 (47,3%) municípios, predominantemente localizados na região Entre Rios (19,8%). A tuberculose, por sua vez, apresentou 56 (25,0%) municípios com essa classificação (20,9 a 45,0 casos/100 mil habitantes), concentrados na mesma região (17,9%). Em relação às doenças negligenciadas em geral, 56 (25,0%) municípios tiveram média de incidência alta (56,8 a 180,4 casos/100 mil habitantes), com maior destaque também na região Entre Rios (19,6%) (Figura 2).

O grupo agregado de doenças tropicais negligenciadas apresentou uma redução temporal nas taxas de prevalência (VPA -2,79%; IC95% -5,24; -0,28), tendo a leishmaniose visceral (VPA -12,26%; IC95% -27,49; -6,71) e a hanseníase (VPA -5,67%; IC95% -9,47; -1,72) como as únicas doenças que também tiveram diminuição das taxas. Em contrapartida, a doença de Chagas (VPA 19,30%; IC95% 5,09; 35,43) e a tuberculose (VPA 2,20%; IC95% 0,59; 3,84) aumentaram a tendência de prevalência (Tabela 2).

**Tabela 2:** Variação percentual anual (VPA) e intervalo de confiança de 95% (IC95%) da prevalência e incidência (100 mil pessoas) das doenças tropicais negligenciadas. Piauí, 2013-2022

	VPA (IC95%)	p-valor	Tendência
Prevalência			
Doenças tropiciais negligenciadas	-2,79 (-5,24; - 0,28)	0,034	Decrescente
Doença de Chagas	19,3 (5,09; 35,43)	0,012	Crescente
Leishmaniose visceral	-12,26 (-17,49; -6,71)	<0,001	Decrescente
Leishmaniose tegumentar	-1,08 (-17,58; 18,72)	0,894	Estacionária
Hanseníase	-5,67 (-9,47; -1,72)	0,011	Decrescente
Tuberculose	2,2 (0,59; 3,84)	0,014	Crescente
Incidência			
Doenças tropiciais negligenciadas	-2,96 (-5,25; 0,62)	0,02	Decrescente
Doença de Chagas	-	-	-
Leishmaniose visceral	-7,88 (-11,18; -4,46)	<0,001	Decrescente
Leishmaniose tegumentar	-1,26 (-15,46; 15,31)	0,855	Estacionária
Hanseníase	-5,75 (-9,65; -1,69)	0,012	Decrescente
Tuberculose	1,57 (-0,16; 3,33)	0,069	Estacionária

Observando-se as taxas de incidência (Tabela 2), também foi identificado que o grupo agregado de doenças tropicais negligenciadas teve redução (VPA -2,96%; IC95% -5,25; -0,62), assim como a leishmaniose visceral (VPA -7,88%; IC95% -11,18; -4,46) e a hanseníase (VPA -5,75%; IC95% -9,65; -1,69).

A Figura 3 apresenta a média da série histórica a respeito do quantitativo de doenças que se sobrepõe nos municípios do Piauí. Essa sobreposição simples é visualizada para doenças de casos que já existem (prevalência) (3A) e apenas para casos novos (incidência) (3B).

**Figura 3:** Sobreposição por ocorrência de agravos das doenças tropicais negligenciadas. Piauí, 2013-2022

Com relação aos casos existentes (3A), apenas Teresina apresentou a sobreposição dos cinco agravos deste estudo. Em contrapartida, Bela Vista do Piauí e Miguel Leão foram classificados como municípios silenciosos, ou seja, aqueles que tiveram classificação zero nos grupos de doenças sobrepostas. Na Figura 3B, que considera casos novos, foi possível verificar que Teresina, Picos e Pedro II foram os municípios que apresentaram a sobreposição de todas as doenças deste estudo. No entanto, Bela Vista do Piauí, Miguel Leão e Vila Nova do Piauí foram classificados como municípios silenciosos. 

## Discussão 

A análise dos dados entre 2013 e 2022 identificou padrões específicos na ocorrência de doenças tropicais negligenciadas no Piauí, caracterizados por uma alta concentração de registros e uma distribuição heterogênea entre as regiões de saúde. Os achados indicam que a hanseníase e a tuberculose se destacam como os agravos mais prevalentes, enquanto a doença de Chagas e a tuberculose apresentaram tendências de aumento nas taxas de prevalência ao longo do período analisado. A sobreposição de múltiplas doenças negligenciadas em determinados municípios, especialmente na capital, Teresina, e em regiões como Entre Rios e Vale do Rio Guaribas sinaliza áreas de maior complexidade epidemiológica no território piauiense.

O perfil epidemiológico observado, com maior acometimento do sexo masculino e da faixa etária de 20-59 anos, evidencia padrões nacionais das doenças negligenciadas, que frequentemente apontam para uma maior exposição ocupacional e para comportamentos de risco entre homens em idade produtiva. O Boletim Epidemiológico das Doenças Tropicais Negligenciadas divulgado pelo Ministério da Saúde em 2024 e alguns estudos nacionais e internacionais, por exemplo, corroboram essa tendência de maior incidência em homens, o que pode refletir diferenças na busca por assistência à saúde. A concentração de casos na região de saúde de Entre Rios, que abriga Teresina, pode ser atribuída à maior densidade populacional e ao papel da capital como centro de referência para diagnósticos e tratamentos de saúde, atraindo pacientes de diversas localidades (5,6,11-13).

A predominância da hanseníase e da tuberculose neste estudo reitera a persistência desses desafios no Piauí. Corroborando os achados desta investigação, estudos recentes sobre o Piauí revelam a manutenção de elevados indicadores para essas doenças, apresentando taxas de detecção em consonância com o cenário de endemicidade que se identificou neste estudo. Similarmente, estudos sobre a tuberculose no Piauí também apontam para a necessidade de atenção contínua. Embora o estado tenha demonstrado avanços em indicadores nos últimos anos, a identificação de áreas endêmicas para hanseníase e tuberculose apontam para a necessidade de estratégias mais eficazes, especialmente pela forte associação desses agravos com condições de pobreza, desigualdades sociais e barreiras no acesso a serviços de saúde. A distribuição espacial desses casos, tanto em áreas urbanas quanto em rurais, reforça a hipótese de que a persistência está ligada não apenas a fatores biológicos, mas também a determinantes sociais e estruturais (14-19).

O perfil epidemiológico da doença de Chagas, que se mostrou mais prevalente na região Vale do Rio Guaribas, é relevante. O aumento na tendência de prevalência desse agravo pode estar relacionado ao aprimoramento da vigilância epidemiológica e da capacidade diagnóstica, especialmente para a fase crônica da doença. Esse fato ressalta a importância de análises temporais cuidadosas ao interpretar dados de notificação compulsória. A maior ocorrência em mulheres, um achado que difere do perfil de outros agravos, é um padrão que também pode ser observado em outras investigações. Esses padrões sugerem que fatores como a exposição domiciliar, a ampliação do rastreamento em contextos específicos, como o pré-natal ou outras fases particulares de cuidado, e a busca por serviços de saúde podem estar envolvidos (20-23).

A alta prevalência da leishmaniose visceral em crianças é outro achado relevante, consistente com estudos anteriores conduzidos no Piauí. A vulnerabilidade infantil à doença é frequentemente atribuída à maior exposição ao vetor em ambientes domésticos e à imaturidade do sistema imunológico. A presença de municípios silenciosos (sem registros de casos) para alguns agravos, como a leishmaniose, levanta a preocupação com a subnotificação ou o subdiagnóstico, o que pode distorcer a real magnitude da doença e impactar a eficácia das ações de vigilância e de controle. Isso pode estar relacionado à insuficiência de recursos, de infraestrutura ou de capacitação técnica para a identificação e a notificação adequada em algumas localidades (14,24,25).

A identificação de municípios com sobreposição de múltiplos agravos, especialmente Teresina, Picos e Pedro II, destaca a complexidade do cenário epidemiológico no Piauí. A concentração de doenças negligenciadas na região de saúde Entre Rios, que inclui a capital do estado e é um polo de referência para estados vizinhos, sugere uma sobrecarga dos serviços de saúde. Embora Teresina possua maior infraestrutura, a alta demanda de pacientes pode comprometer a capacidade de resposta e a eficácia das ações de vigilância e de tratamento na região. A análise espacial e temporal, ao identificar as áreas críticas e as tendências das doenças, é fundamental para o planejamento e a alocação de recursos, contribuindo para uma atuação mais eficaz do Sistema Único de Saúde (26,27).

A tendência de redução das taxas de prevalência e de incidência do grupo agregado de doenças negligenciadas, bem como da leishmaniose visceral e da hanseníase, é um indicativo de que as ações de controle e de diagnóstico precoce estão produzindo resultados positivos, ainda que de forma heterogênea. Em contraste, o aumento observado nas taxas da doença de Chagas e da tuberculose exige uma reavaliação das estratégias vigentes e a adaptação às mudanças nos perfis epidemiológicos.

Este estudo ressalta a importância da intersetorialidade e da implementação de políticas públicas que vão além do setor da saúde para o enfrentamento das doenças tropiciais negligenciadas. O Programa Brasil Saudável, lançado pelo Ministério da Saúde em 2023, exemplifica uma abordagem promissora, ao integrar ações interministeriais voltadas à melhoria das condições de vida e de trabalho da comunidade. A aplicação de estratégias regionais e interministeriais, conforme proposto por essa iniciativa, é crucial para mitigar o impacto desses agravos e promover a equidade em saúde no Piauí. O fortalecimento da rede de Atenção Primária e a descentralização do atendimento são essenciais para garantir o acesso universal a diagnóstico e tratamento, especialmente em territórios mais vulnerabilizados.

Este estudo possui algumas limitações, visto que a utilização de dados secundários pode levar a problemas de subnotificação e de duplicidade de registros, o que pode introduzir vieses nos cálculos e comprometer a precisão das estimativas de incidência e de prevalência. A análise espacial, por sua vez, pode não captar plenamente as dinâmicas locais de transmissão, especialmente em municípios com menor capacidade de vigilância epidemiológica. Apesar dessas limitações, os dados analisados nesta investigação oferecem um panorama relevante da carga e de distribuição das doenças tropicais negligenciadas no estado, subsidiando ações estratégicas de saúde pública.

## Data Availability

O conjunto completo de dados utilizados nesta pesquisa está disponível no repositório Open Science Framework (OSF), disponível em: https://osf.io/38u5y/ (28).
